# Extension of Collagen Deposition in COVID-19 Post Mortem Lung Samples and Computed Tomography Analysis Findings

**DOI:** 10.3390/ijms22147498

**Published:** 2021-07-13

**Authors:** Lorenzo Ball, Emanuela Barisione, Luca Mastracci, Michela Campora, Delfina Costa, Chiara Robba, Denise Battaglini, Marco Micali, Federico Costantino, Giuseppe Cittadini, Nicolò Patroniti, Paolo Pelosi, Roberto Fiocca, Federica Grillo

**Affiliations:** 1 Department of Surgical Sciences and Integrated Diagnostics, University of Genoa, 16126 Genoa, Italy; luca.mastracci@unige.it (L.M.); kiarobba@gmail.com (C.R.); m.micali@hotmail.it (M.M.); fede.tino3@gmail.com (F.C.); npatroniti@gmail.com (N.P.); ppelosi@hotmail.com (P.P.); fiocca@unige.it (R.F.); federica.grillo@unige.it (F.G.); 2 Anesthesia and Intensive Care, San Martino Policlinico Hospital, IRCCS for Oncology and Neurosciences, 16132 Genoa, Italy; battaglini.denise@gmail.com; 3 Interventional Pulmonology Unit, San Martino Policlinico Hospital, IRCCS for Oncology and Neurosciences, 16132 Genoa, Italy; emanuela.barisione@hsanmartino.it; 4 Anatomic Pathology Unit, San Martino Policlinico Hospital, IRCCS for Oncology and Neurosciences, 16132 Genoa, Italy; 5 Surgical Pathology Unit, Santa Chiara Hospital, Provincial Agency for Health Services, 38122 Trento, Italy; michela.campora@apss.tn.it; 6 Molecular Oncology and Angiogenesis Unit, San Martino Policlinico Hospital, IRCCS for Oncology and Neurosciences, 16132 Genoa, Italy; delfina.costa@hsanmartino.it; 7 Radiology Department, San Martino Policlinico Hospital, IRCCS for Oncology and Neurosciences, 16132 Genoa, Italy; giuseppe.cittadini@hsanmartino.it

**Keywords:** COVID-19, fibrosis, collagen, computed tomography

## Abstract

Lung fibrosis has specific computed tomography (CT) findings and represents a common finding in advanced COVID-19 pneumonia whose reversibility has been poorly investigated. The aim of this study was to quantify the extension of collagen deposition and aeration in postmortem cryobiopsies of critically ill COVID-19 patients and to describe the correlations with qualitative and quantitative analyses of lung CT. Postmortem transbronchial cryobiopsy samples were obtained, formalin fixed, paraffin embedded and stained with Sirius red to quantify collagen deposition, defining fibrotic samples as those with collagen deposition above 10%. Lung CT images were analyzed qualitatively with a radiographic score and quantitatively with computer-based analysis at the lobe level. Thirty samples from 10 patients with COVID-19 pneumonia deceased during invasive mechanical ventilation were included in this study. The median [interquartile range] percent collagen extension was 6.8% (4.6–16.2%). In fibrotic compared to nonfibrotic samples, the qualitative score was higher (260 (250–290) vs. 190 (120–270), *p* = 0.036) while the gas fraction was lower (0.46 (0.32–0.47) vs. 0.59 (0.37–0.68), *p* = 0.047). A radiographic score above 230 had 100% sensitivity (95% confidence interval, CI: 66.4% to 100%) and 66.7% specificity (95% CI: 41.0% to 92.3%) to detect fibrotic samples, while a gas fraction below 0.57 had 100% sensitivity (95% CI: 66.4% to 100%) and 57.1% specificity (95% CI: 26.3% to 88.0%). In COVID-19 pneumonia, qualitative and quantitative analyses of lung CT images have high sensitivity but moderate to low specificity to detect histopathological fibrosis. Pseudofibrotic CT findings do not always correspond to increased collagen deposition.

## 1. Introduction

Long-term consequences of severe acute respiratory syndrome coronavirus 2 (SARS-Cov-2) associated with interstitial pneumonia and its complications, including acute distress respiratory syndrome (ARDS), have been recently highlighted [1,2]. Patients with severe COVID-19 pneumonia typically meet the clinical criteria for ARDS [3] and specific pathophysiological aspects have been described [4,5]. In particular, concerns regarding the fibrotic evolution of lung disease in COVID19 survivors are being raised [6,7], and a specific fibrotic phenotype with peculiar histopathological findings and radiological morphology has been described [8].

Postmortem studies have identified COVID-19-related lung injury characterized by diffuse alveolar damage (DAD) with an early exudative phase with edema and hyaline membranes, followed by an organizing phase with loose organizing fibrosis and type II pneumocyte hyperplasia, and a final fibrotic stage found only in patients with longer duration of disease [9,10]. The disease spectrum has also been described in postmortem cryobiopsy lung samples [11,12,13]. Chest computed tomography (CT) is widely used in the clinical management of patients with acute respiratory failure [14], including in COVID-19 [15]. Radiographic signs compatible with lung fibrosis have been described both in the acute phase of COVID-19 [16] and during the follow up [17]. Collagen deposition is a hallmark of lung fibrosis [18], and its quantification could help define the evolutive characteristics of COVID-19 patients. However, its association with CT findings has not yet been described, and previous CT studies in ARDS have poorly explored the fibrotic phase [8,19].

The aim of this study is to quantify the extension of collagen deposition in postmortem cryobiopsies of critically ill COVID-19 patients and to describe sensitivity and specificity of qualitative and quantitative analyses of lung CT. We hypothesized that collagen deposition would be associated with qualitative and quantitative computed tomography parameters.

## 2. Results

### 2.1. Patients and Samples Inclusion

Ten COVID-19 patients were included in this analysis, of which eight were previously included in another study [11]; clinical characteristics are reported in Table 1. All patients had received a CT scan, and the median time from CT scan to death was 4 (3–4) days. All patients except one had received a course of methylprednisolone during t heir hospital stay.

Due to technical difficulties related to the cryobiopsy procedure, not all patients had an available sample for each lung lobe: six (60%) had available samples from the LLL, seven (70%) from the LUL and RML, eight (80%) from the RLL and four (40%) from the RUL. Two samples were judged inadequate for analysis due to mechanical deformation during the sampling process, for a total of 30 valid samples from 10 patients included in this study.

### 2.2. Quantitative Histopathological Analysis

**Figure 1:**
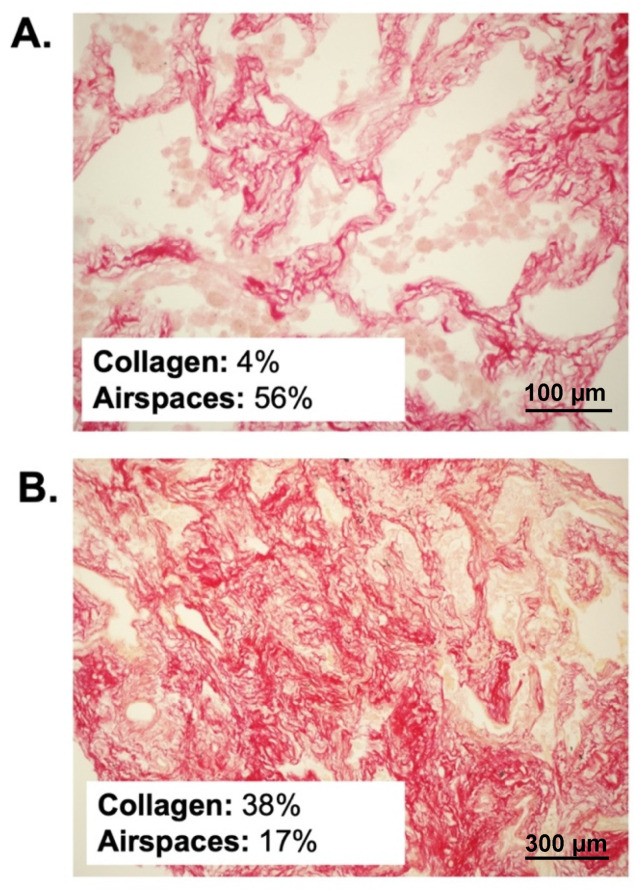
Histopathological samples stained with Sirius red in two representative patients. (**A**) illustrates a patient with early disease, normal percent collagen amount and normal alveolar airspaces. (**B**) illustrates a patient with advanced disease, parenchymal disruption, pathological collagen deposition and extensive loss of aeration.

The median sample surface included in the analysis was 1.6 (0.9–2.4) mm^2^. Two representative samples are illustrated in Figure 1.

**Figure 2:**
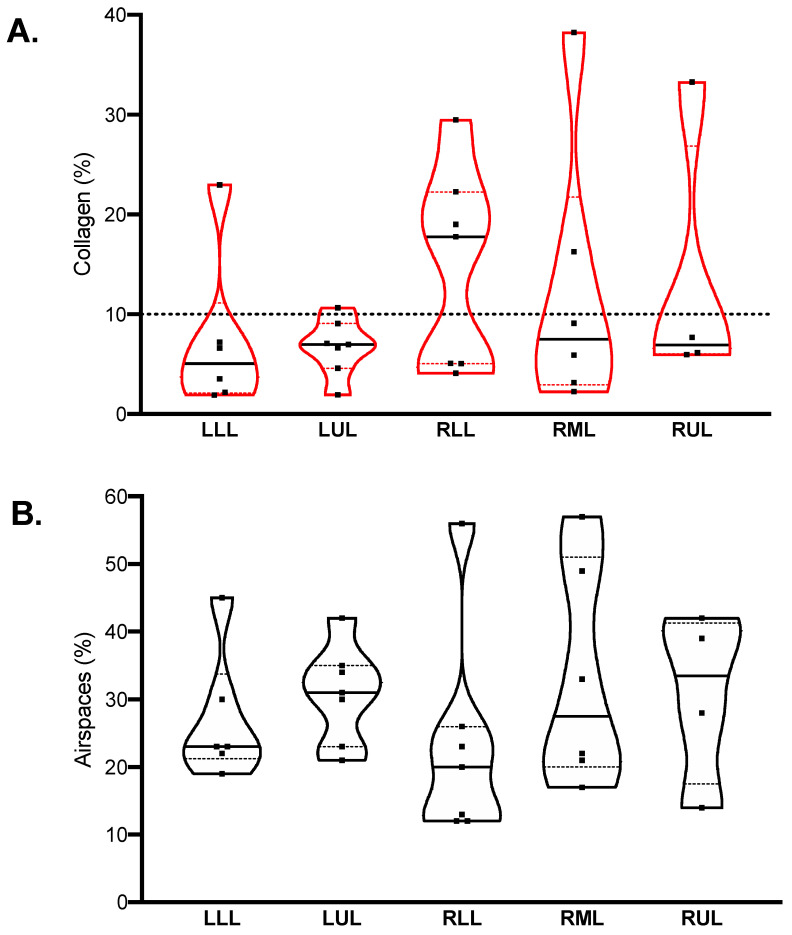
Quantification of collagen (**A**) and airspaces (**B**) in Sirius red-stained samples. Violin plots represent distributions, squares represent individual patient data, thick lines medians, thin dotted lines quartiles. The dotted horizontal line in (panel (**A**)) represents the threshold defining fibrotic samples (collagen > 10%).

The median percent collagen extension was 6.8 (4.6–16.2)%, while airspace extension was 25 (21–35)%. Their distribution in different lobes is illustrated in Figure 2. In the healthy historical cohort, collagen extension was 5.9 (5.0–7.4)% (mean 5.9%, standard deviation 1.9%); therefore, the cut-off for defining fibrotic samples was set to 10%. Nine samples were classified as fibrotic, while 21 were nonfibrotic.

### 2.3. Qualitative CT Assessment and Quantitative CT Analysis

**Figure 3:**
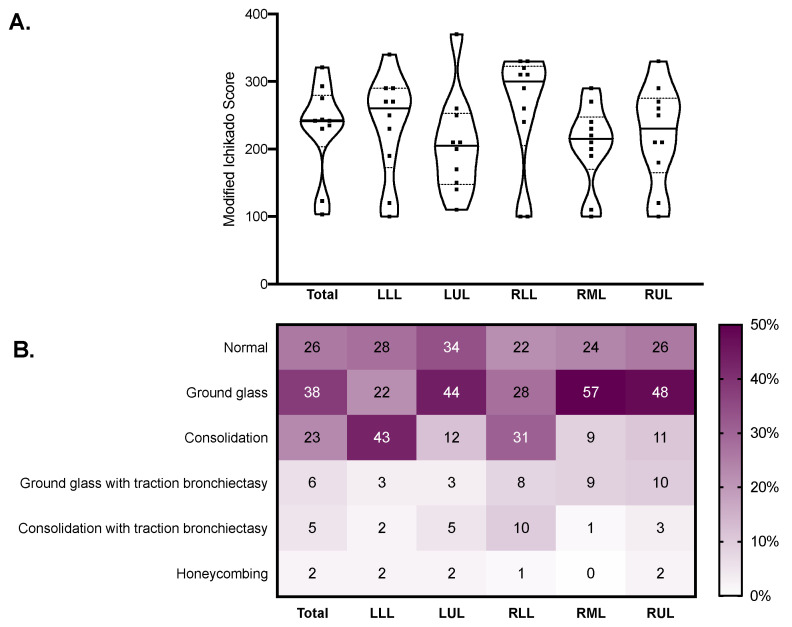
Qualitative CT analysis results in the whole lungs (total) and in each lobe. Violin plots in (**A**) represent distributions, squares represent individual patient data, thick lines medians, thin dotted lines quartiles. The heatmap in (**B**) reports the mean estimated percent extension of each CT finding. CT: computed tomography; LLL: left lower lobe; LUL: left upper lobe; RLL: right lower lobe; RML: right middle lobe; RUL: right upper lobe.

**Figure 4:**
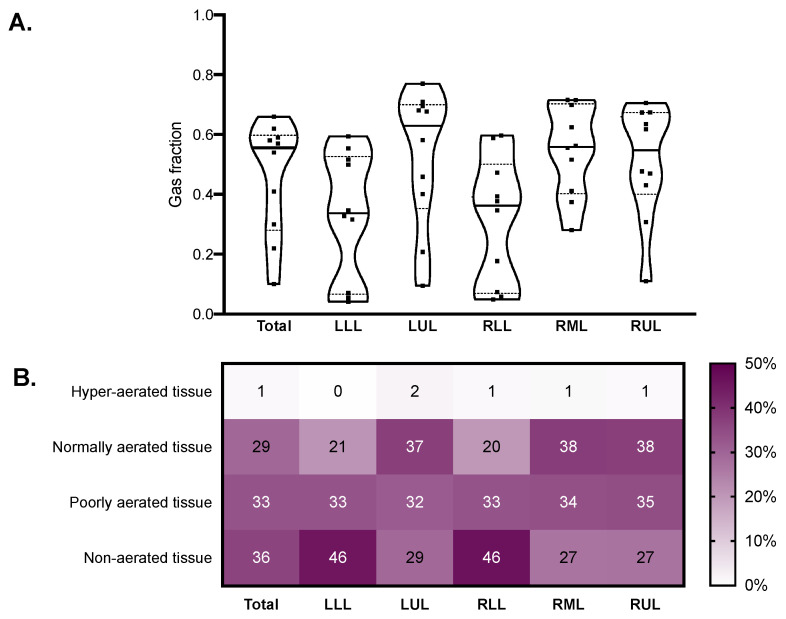
Quantitative CT analysis results in the whole lungs (total) and in each lobe. Violin plots in (**A**) represent distributions, squares represent individual patient data, thick lines medians, thin dotted lines quartiles. The heatmap in (**B**) reports the mean extension of each quantitative CT aeration compartment expressed as percent of the total lobe tissue. CT: computed tomography; LLL: left lower lobe; LUL: left upper lobe; RLL: right lower lobe; RML: right middle lobe; RUL: right upper lobe.

The median modified Ichikado score was 242 (230–275) and was higher in fibrotic versus nonfibrotic samples (260 (250–290) vs. 190 (120–270), *p* = 0.036). As illustrated in Figure 3, the score was higher in the lower lobes, where consolidation and traction bronchiectasis were more represented. Honeycombing was reported in seven samples from two patients. The median gas fraction was 0.56 (0.30–0.59), being lower in fibrotic versus nonfibrotic samples (0.46 (0.32–0.47) vs. 0.59 (0.37–0.68), *p* = 0.047). Figure 4 illustrates the distribution of the gas fraction and the extension of aeration compartments. Detailed results of the quantitative CT analysis are reported in Table 2.

### 2.4. Receiver Operating Characteristics Analysis

The results of the receiver operating characteristics analysis are reported in Table 3. An AUC of 0.746 (95% CI from 0.572 to 0.920) was observed for the modified Ichikado score. A score above 230 had high sensitivity (100%, 95% CI from 66.4% to 100%) but moderate specificity (66.7%, 95% CI from 41.0% to 92.3%) to detect fibrosis defined as collagen deposition > 10%. Consolidation with traction bronchiectasis and honeycombing was highly specific for fibrosis but had very low sensitivity. The AUC for the gas fraction was 0.725 (95% CI from 0.547 to 0.903) and a value below 0.57 had high sensitivity (100%, 95% CI from 66.4% to 100%) but moderate specificity (57.1%, 95% CI from 26.3% to 88.0%) to detect fibrotic samples.

### 2.5. Associations between Qualitative and Quantitative CT Analysis and Histopathology

**Figure 5:**
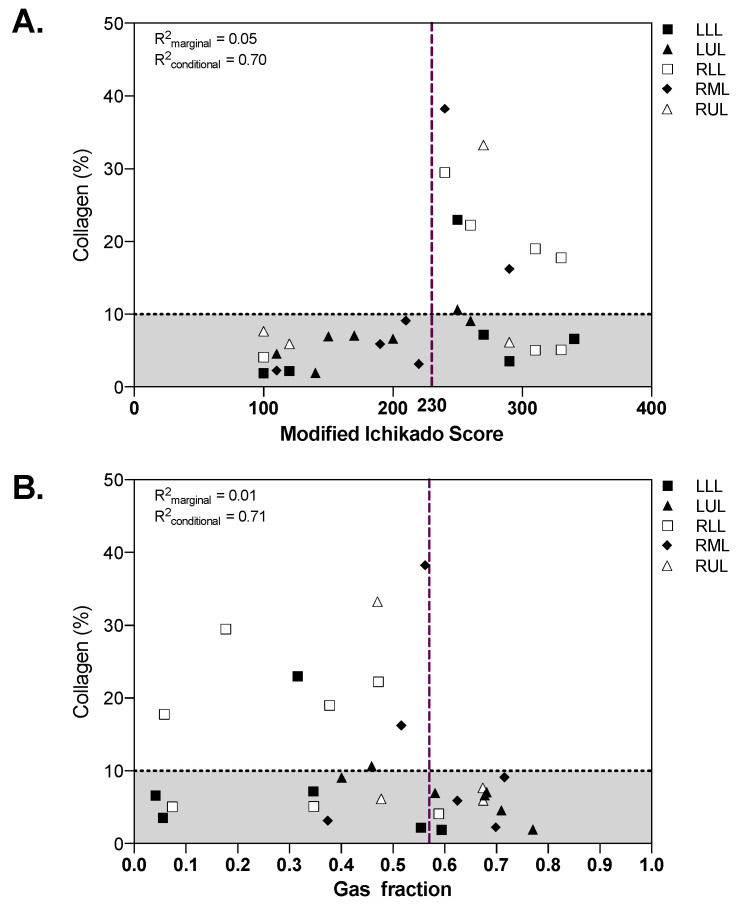
Qualitative (**A**) and quantitative (**B**) CT analysis as function of collagen percent extension. The dotted horizontal line in panel A represents the threshold defining fibrotic samples (collagen > 10%), vertical dashed lines represent the cut-off identified in the receiver operating characteristics analysis. The marginal *R*^2^ describes the proportion of variance explained by the CT parameter alone, the conditional *R*^2^ the proportion of variance explained by both the CT parameter and the random effect (patient). CT: computed tomography; LLL: left lower lobe; LUL: left upper lobe; RLL: right lower lobe; RML: right middle lobe; RUL: right upper lobe.

Both the modified Ichikado score (*R*^2^*_marginal_* = 0.05, *R*^2^*_conditional_* = 0.70) and the gas fraction (*R*^2^*_marginal_* = 0.01, *R*^2^*_conditional_* = 0.71) were associated with the amount of collagen in lung samples (Figure 5). While all fibrotic samples with collagen above 10% had high radiographic score and low gas fraction, nonfibrotic samples showed a wide range of radiographic scores and gas fractions. The extension of airspaces was correlated with the CT gas fraction (*R*^2^*_marginal_* = 0.11, *R*^2^*_conditional_* = 0.29).

## 3. Discussion

In critically ill COVID-19 patients deceased during invasive mechanical ventilation, we found that: (1) lung collagen amount ranged from normal to extremely high; (2) qualitative and quantitative CT analysis had weak correlation with collagen amount in postmortem lung samples; (3) qualitative and quantitative CT parameters had high sensitivity but moderate specificity to detect histopathological fibrosis.

In the present study the amount of collagen deposition in postmortem samples of patients with COVID-19 pneumonia was quantitatively evaluated by using specific staining and quantitative histopathologic image analysis. The correlations between qualitative and quantitative lung CT findings were explored, observing that CT-derived parameters were weakly correlated with the amount of collagen fibers. The analysis was conducted at the lobe level, thus considering the high spatial heterogeneity of the disease. The lung CTs were performed near to the date of death, allowing a reliable comparison between lung imaging and histology. The use of postmortem cryobiopsies allowed an analysis of the lung immediately after death and during the application of positive pressure to the airways to maintain lung aeration, thus minimizing the artifacts at histopathological analysis that might occur during conventional autoptic exams. 

Evidence at hospital discharge shows that nearly half of survivors of COVID-19 have impaired lung diffusing capacity for carbon monoxide, a quarter have reduced total lung capacity, and this is correlated with the severity of disease [20]. Furthermore, 55% to 80% of COVID-19 patients show impaired pulmonary function and radiologic abnormalities three months after ICU discharge, while 21% show fibrotic patterns at the computed tomography (CT) [17,21,22]. It is still unclear whether patients will show recovery over time or irreversible fibrosis will develop. Our findings suggest that radiological signs of fibrosis at the CT scan might not always be associated with increased collagen deposition; one might speculate that these pseudo-fibrotic CT findings with low amounts of collagen could be reversible and that the respiratory function might improve with time after recovery. In ARDS from causes other than COVID-19, most survivors show progressive resolution of pulmonary function abnormalities, while around one quarter develop a restrictive pattern [23], which tends to stabilize over time, different from what is seen in idiopathic pulmonary fibrosis [24,25]. Fibrotic pulmonary remodeling has also been described after SARS pneumonia, with 15–20% of survivors showing reduced lung functional capacity within the first year [26], and 5% having stable interstitial abnormalities at 15 years [27]. This study provides insights on the molecular mechanisms of lung damage in COVID-19 and provides a basis for clinical studies investigating the reversibility of CT and functional parameters in survivors with suspect fibrotic evolution. Our findings might help to clarify the role of CT imaging analysis in assessing fibrotic evolution in COVID-19; this could have implications in studies assessing clinical evolution after hospital discharge and in monitoring the effects of drugs with antifibrotic properties in the acute or post acute phase [28]. Of notice, we observed that ground glass opacities, consolidation, and ground glass opacities with traction bronchiectasis, had specificity ranging from 42.9% to 76% to detect samples with increased collagen deposition. This suggests that the presence of these findings at the CT scan could be part of the evolution of the pathology not necessarily reflecting proliferative fibrosis. Quantitative CT analysis, as compared to qualitative scoring, resulted in similar performance in the detection of increased collagen. This might be explained by the fact that quantitative CT analysis mainly relies on the relative amount of gas and lung tissue contained in each voxel [29], not taking into account the specific patterns observed in lung fibrosis.

The high sensitivity, but moderate specificity, of CT findings observed in our study could explain in part the finding that lung function in severe COVID-19 survivors may return to normal or significantly improve over time [17,22,30]. Notwithstanding this, even a relatively small percentage of post COVID-19 nonprogressive fibrosis, given the sheer numbers of affected individuals, could result in considerable morbidity. A recent study reported a 24% incidence of persistent CT alterations at 12 months [31]. What is known up till now from COVID-19 autoptic series is that initial lung pathology is characterized by florid diffuse alveolar damage associated with immune-mediated thrombotic microangiopathy, while latter stages are characterized by an organizing phase with fibroblastic proliferation and a late stage with fibrosis and possible honeycombing, commencing generally three weeks after onset of symptoms [32,33]. However, no study has until now evaluated the pathological aspects of COVID-19 survivors with fibrotic sequelae on CT scans; evaluation of which could be performed using lung cryobiopsy procedures (and which is not available for SARS either [27].

Our study has limitations that should be addressed. First, the sample size was small. Our cohort, however, included a wide range of lung involvement and collagen deposition, representative of a high proportion of the general COVID-19 population. Second, analyses were conducted at the lobe level, and correlations should be interpreted cautiously since multiple samples were collected from each patient. This was necessary due to the high heterogeneity of lung lesions observed in COVID-19, and robust statistical analyses to compensate for repeated measurements were adopted in this study. Third, a single stain to assess fibrosis, unable to distinguish between different collagen types, was used. This allowed development of a simple method to quantify overall collagen deposition, representative of the extension of lung fibrosis. Finally, the use of a cryobiopsy technique, though enabling the sampling of extremely well-preserved tissue, has the limitation of being small in size, making lesional heterogeneity a possible limit in the correlation between imaging and histology.

## 4. Methods

This study was conducted in a university-affiliated hospital in Genoa, Italy. The ethics review board approved the protocol of the study (Comitato Etico Regione Liguria, protocol n. 144/2020-DB, id 10460) and consent was obtained by telephone by the next of kin and documented on clinical records by the treating intensivist. Patients deceased in the ICU while receiving invasive mechanical ventilation were included from 6 April to 16 June 2020. Exclusion criteria were age <18 years, previous chronic pulmonary conditions, pregnancy and unavailability of the research staff.

### 4.1. Postmortem Sampling Procedure

Postmortem transbronchial cryobiopsy samples were obtained as previously described [11]. After patient death, patient lung aeration was maintained by applying a constant positive airway pressure equal to the positive end-expiratory pressure received before death. As per national regulations, death was ascertained with a continuous 20-min flat electrocardiogram, while the sampling procedure was initiated within 30 min from death. Cryobiopsies were obtained with a 1.7 mm cryoprobe (ErbeCryo^®^, Erbe Elektromedizin GmbH, Tuebingen, Germany) inserted through the operative channel of a single use flexible video-bronchoscope (Ambu^®^ aScope^TM^, Ambu, Ballerup, Denmark) and operated for 10–11 s. Biopsies were collected and immediately fixed in 10% formalin for each lobe: right lower lobe (RLL), right middle lobe (RML), right upper lobe (RUL), left lower lobe (LLL) and left upper lobe (LUL).

### 4.2. Histologic Analysis and Quantification of Collagen Deposition and Aeration on Lung Samples

From formalin-fixed paraffin-embedded tissue blocks, 4-micron thick microtome sections were stained with Sirius red as per the manufacturer’s instructions (DiaPath SPA, Bergamo, Italy). All histopathological analyses were performed by two experienced pathologists (FG and LM), blinded to the radiographic findings, adapting the methodology from previous experiences in liver fibrosis [34,35]. Sirius red images were acquired using the 40× objective of an Aperio AT2 scanner and Aperio Image-Scope software 9.1 (Leica Biosystems, Nussloch, Germany). The samples were acquired if they met prespecified adequacy criteria: dimensions of the samples, surface area and percentage of lung parenchyma present in each biopsy [36]. Samples were manually revised, and three representative regions of interest (ROIs) from a single sample from each lobe were selected, manually excluding airway walls and vessels with diameter > 200 μm. Extensions of stained areas and airspaces were computed with automated image analysis (Aperio color deconvolution algorithm version 9.1, Leica Biosystems, Nussloch, Germany) [37]. After identification of a reference point for Sirius red color in each sample, stained areas were defined based on the default threshold levels of the image analysis software; areas were classified as collagen with intensity below 114 (intense red staining) and as alveolar airspaces with intensity above 240 (clear regions), with intermediate values representing lung structures not stained by Sirius red. The percent tissue collagen and airspaces extension in each ROI were computed as follows:

(1) $$Collagen\;{\left(\%\right)}_{ROI}=100\;\cdot \;\frac{Surface_{Collagen}}{Surface_{Lung\;tissue}}=\;100\;\cdot \;\frac{Surface_{Collagen}}{Surface_{ROI}-Surface_{Airspaces}}$$

(2) $$Airspaces\;{\left(\%\right)}_{ROI}=100\;\cdot \;\frac{Surface_{Airspaces}}{Surface_{ROI}}$$

Percent collagen and airspaces extension of each sample were obtained as averages of the three ROIs. Lung samples were classified as fibrotic if their collagen deposition was above two standard deviations from the mean of a reference value obtained from three healthy lung samples of a historical cohort. The historical cohort included samples of histologically normal lung parenchyma obtained from resection specimens (nonlesional lung tissue far from pathologic areas).

### 4.3. Computed Tomography Acquisition and Segmentation

The latest CT scan available for each patient was retrospectively collected. All scans were performed during end-expiratory breath hold using a high-resolution multidetector scanner (Siemens Definition Flash, 128 slices, Erlangen, Germany), and reconstructed at a slice thickness of 1.25 or 0.75 mm with a sharp B80f convolution kernel. All scans were manually segmented using open-source software (ITKSnap 3.8.0, http://www.itksnap.org, accessed 20 May 2021) excluding large vessels and airways. The segmentation was conducted separately for each lung lobe.

### 4.4. Computed Tomography Qualitative Assessment

The extension of lung disease was assessed using a radiographic score based on the Ichikado score, modified to be computed at the lobar level instead of in six topographic lung regions. The Ichikado score was initially proposed for grading [38] and prognostication [39] of acute interstitial pneumonia and later validated in ARDS [19]. For each lobe the extension of six CT patterns representing evolutive pathologic phases was estimated, rounded to the nearest 10%. Scores were classified as follows: score of 1, normal; score of 2, ground-glass opacities; score of 3, consolidation; score of 4, ground-glass attenuation plus traction bronchiectasis; score of 5, consolidation plus traction bronchiectasis, and score of 6, honeycombing. The modified Ichikado score of each lobe was calculated multiplying the percentage extension by the score value of each compartment. The whole-lung score was computed, averaging the score of the five lobes.

### 4.5. Computed Tomography Quantitative Analysis

The quantitative analysis of lung images was conducted using custom-made Matlab scripts (Mathworks Inc., Natick, MA, USA), using established methods. Lung density was considered proportional to the gas versus tissue fraction contained within each voxel, approximating tissue density as 1 g/mL [29]. The extent of four compartments was measured, based on their degree of aeration based on standard Hounsfield units (HU) thresholds: −900 HU, −500 HU and −100 HU, to define hyperaerated, normal, poorly aerated, and nonaerated areas, respectively [40]. The gas fraction (*G_f_*) was computed as:

(3) $$G_{f}=\frac{Gas\;volume}{Total\;volume}=\frac{H}{\begin{array}{c} -1000 \end{array}}$$

where *H* is CT attenuation in HU. Analyses were conducted on the entire lungs and at the lobar level.

### 4.6. Statistical Analysis

The primary endpoint of the study was the qualitative radiographic score. Secondary endpoints included extension of collagen deposition and qualitative and quantitative CT analysis parameters. An a priori sample size calculation was not feasible due to the lack of data on the extension of collagen deposition in COVID-19 patients. Data are reported as median [interquartile range], if not otherwise specified. Receiver operating characteristics (ROC) curves were used to evaluate the diagnostic ability of selected variables to identify fibrotic histopathological samples with increased collagen deposition. For each CT parameter, the area under the curve (AUC) was computed and the sensitivity and specificity were calculated for the cut-off value identified with the Youden method. Confidence intervals for sensitivity and specificity were corrected for repeated measures using the variance inflation factor method [41]. As a sensitivity analysis, associations were further investigated with mixed linear models using patient as random effect with a random intercept and radiographic findings, and lung lobe as fixed effects, reporting the marginal and conditional *R*^2^ values [42] as diagnostics. The marginal *R*^2^ describes the proportion of variance explained by the covariate alone, and the conditional *R*^2^ the proportion of variance explained by both covariates and random factors. All statistical analyses were performed in R 4.0.2 (The R foundation) using the {lmerTest}, {ROCit} and {MuMIn} packages. Statistical significance was assumed at two-tailed *p* < 0.05.

## 5. Conclusions

In COVID-19 pneumonia, qualitative and quantitative analysis of lung CT images have high sensitivity but moderate to low specificity to detect histopathological fibrosis. Pseudofibrotic CT findings do not always correspond to increased collagen deposition.

## Figures and Tables

**Table 1:** Population description.

	Overall Population (*n* = 10)
General characteristics	
Age, median (IQR)	72 (60–77)
Sex (male), *n* (%)	7 (70)
Time from onset of symptoms to ICU admission, median (IQR), days	11 (8–16)
Time from hospital admission to ICU admission, median (IQR), days	9 (3–13)
Time from ICU admission to death, median (IQR), days	17 (10–27)
Time from last CT scan to death, median (IQR), days	4 (3–4)
Comorbidities	
Hypertension, *n* (%)	6 (60)
Diabetes, *n* (%)	1 (10)
Smoker, *n* (%)	2 (20)
Ischemic cardiopathy, *n* (%)	1 (10)
Specific treatments received	
Methylprednisolone, *n* (%)	9 (90)
Tocilizumab, *n* (%)	3 (30)
Hydroxychloroquine, *n* (%)	8 (80)
Leading cause of death	
Refractory hypoxemia, *n* (%)	7 (70)
Cardiocirculatory failure, *n* (%)	3 (30)
Oxygenation	
PaO_2_/FiO_2_ ratio at ICU admission, median (IQR), mmHg	173 (112–310)
PaO_2_/FiO_2_ ratio the day of death, median (IQR), mmHg	77 (55–99)

IQR: interquartile range; ICU: intensive care unit; CT: computed tomography.

**Table 2:** Quantitative computed tomography analysis.

Variable	Total	Left Lower Lobe	Left Upper Lobe	Right Lower Lobe	Right Middle Lobe	Right Upper Lobe
Volume (mL), median (IQR)	2682 (2131–3554)	410 (395–641)	708 (518–1048)	504 (468–594)	269 (238–395)	679 (503–892)
Attenuation (HU), median (IQR)	−555 (−595–−301)	−337 (−517–−71)	−629 (−696–−401)	−362 (−472–−73)	−559 (−699–−412)	−547 (−674–−431)
Weight (g), median (IQR)	1460 (1239–1917)	301 (260–376)	336 (305–375)	355 (285–510)	139 (115–167)	304 (263–419)
Vgas (mL), median (IQR)	1389 (701–2278)	132 (29–323)	414 (225–744)	176 (34–218)	148 (102–263)	374 (236–538)
Gas Fraction, median (IQR)	0.56 (0.30–0.59)	0.34 (0.07–0.52)	0.63 (0.40–0.70)	0.36 (0.07–0.47)	0.56 (0.41–0.70)	0.55 (0.43–0.67)
Hyperaerated tissue (%), median (IQR)	0.6 (0.2–1.3)	0.2 (0.1–0.3)	1.1 (0.2–2.4)	0.1 (0.1–0.4)	0.7 (0.3–1.5)	0.6 (0.2–1.1)
Normally aerated tissue (%), median (IQR)	28.7 (12.6–49.6)	14.1 (4.1–40.3)	43.1 (20.7–53.0)	16.4 (2.4–36.3)	41.3 (17.2–57.5)	37.8 (19.4–60.4)
Poorly aerated tissue (%), median (IQR)	33.7 (30.6–36.0)	31.4 (28.8–37.0)	32.1 (26.2–35.4)	29.4 (27.6–37.3)	34.5 (28.8–40.7)	33.6 (31.0–40.2)
Nonaerated tissue (%), median (IQR)	34.2 (16.3–53.8)	49.4 (23.2–69.4)	25.4 (13.3–41.0)	47.2 (26.0–70.3)	24.0 (14.2–41.5)	22.9 (11.7–45.7)

IQR: interquartile range; HU: Hounsfield units.

**Table 3:** Receiver operating characteristic of qualitative and quantitative CT parameters for detection of fibrotic lung samples (collagen > 10%).

Parameter	AUC (95% CI)		Cut-Off	Sensitivity, % (95% CI)		Specificity, % (95% CI)	
Qualitative Analysis							
Normal lung (estimated %)	0.742	(0.567–0.919)	<25	100	(66.4–100)	47.6	(10.4–84.8)
Ground glass opacities (estimated %)	0.648	(0.451–0.845)	>25	77.8	(45.1–100)	52.4	(22.0–82.7)
Consolidation (estimated %)	0.685	(0.496–0.875)	>5	100.0	(66.4–100)	42.9	(10.1–75.6)
Ground glass with traction bronchiectasis (estimated %)	0.688	(0.461–0.915)	>5	55.6	(29.5–81.6)	76.2	(55.5–96.9)
Consolidation with traction bronchiectasis (estimated %)	0.521	(0.288–0.754)	>15	11.1	(0.0–33.6)	95.2	(87.0–100)
Honeycombing (estimated %)	0.563	(0.329–0.798)	>5	22.2	(0.0–51.5)	90.5	(74.0–100)
Modified Ichikado score	0.746	(0.572–0.920)	>230	100.0	(66.4–100)	66.7	(41.0–92.3)
Lobe weight (g)	0.624	(0.389–0.859)	>263	77.8	(62.9–92.7)	52.4	(32.4–72.3)
Gas fraction	0.725	(0.547–0.903)	<0.57	100.0	(66.4–100)	57.1	(26.3–88.0)
Hyperaerated tissue (%)	0.762	(0.584–0.940)	<0.2	77.8	(57.0–98.6)	81.0	(62.3–99.6)
Normally aerated tissue (%)	0.667	(0.465–0.868)	<40	88.9	(68.9–100)	61.9	(29.5–94.3)
Poorly aerated tissue (%)	0.661	(0.432–0.891)	>41	44.4	(25.4–63.5)	95.2	(86.5–100)
Nonaerated tissue (%)	0.593	(0.356–0.829)	>24	77.8	(37.9–100)	57.1	(24.0–90.3)

CT: computed tomography; AUC: area under the curve; CI: confidence interval; PPV: positive predictive value; NPV: negative predictive value. Confidence intervals of sensitivity and specificity are adjusted for clustered measurements using the variance inflation factor method.

## Data Availability

Datasets are available from the corresponding author upon reasonable request.
